# Treatment of the so-called idiopathic scoliosis by physiotherapy - incorrect or correct – examples of both group of children

**DOI:** 10.1186/1748-7161-9-S1-P10

**Published:** 2014-12-04

**Authors:** Tomasz Karski, Jacek Karski

**Affiliations:** 1 Vincent Pol University, Lublin, Poland; 2 Department of Paediatric Orthopaedic and Rehabilitation, Medical University, Lublin, Poland

## Introduction

The biomechanical aetiology of so-called idiopathic scoliosis called AIS is described in years 1995 – 2007 (T. Karski) and presented since 2002 in many Congresses and Symposia.

## Material

In 2012 the whole material gathered 1950 cases. Patients were 2 to 60 years old.

Explanation of biomechanical aetiology of scoliosis in point

1. “Syndrome of contractures”[SofC] (Siebenersyndrom) according to Prof.Hans Mau.

2. Asymmetry in movement of hips connected with SofC. Limited movement of the right hip.

3. Influence on spine comes by walking (gait) and because of habit of permanent standing ‘at ease’ on the right leg.

New classification as input for physiotherapy

There are three groups and four types (T. Karski 2001 – 2004).

1) “S” I etiopathological (epg) scoliosis. Influenced by the “gait” and the permanent “standing at ease on the right leg”.

2)

a. 2A/“C” II/A epg scoliosis – Influenced by the permanent “standing at ease on the right leg”.

b. 2B/ “S” II/B epg scoliosis - Influenced by the permanent “standing at ease on the right leg”, plus - laxity of joints or/and incorrect exercises in previous treatment.

3) “I” III epg scoliosis. Influenced by the “gait” only.

## Physiotherapy

All previous extensions, its mean “muscles strengthening exercises” were incorrect and caused only bigger curves and made the spine more stiff. All stretching exercises are proper for treatment and for prophylaxis

## Conclusions

1. All scientists and all Institutions engaged with scoliosis should learn about “biomechanical reasons in development of scoliosis”.

2. All orthopaedic surgeons, rehabilitations and physiotherapies should be introduced to the new conception of treatment and of causal prophylaxis in children with so–called idiopathic scoliosis using the own material.
